# Beyond Stenosis: Prevalence and the Characteristics of Nonstenotic Carotid Plaques in Embolic Stroke of Undetermined Source

**DOI:** 10.1159/cee/acdag001

**Published:** 2026-08-04

**Authors:** Manuraj Natarajan, Adarsh Anil Kumar, Asish Vijayaraghavan, Santhosh Kannoth, Sapna Erat Sreedharan, Arya Devi Karuthedathu mana Sanal Kumar, Padmavathy Narayanan Sylaja

**Affiliations:** a Comprehensive Stroke Care Program, Department of Neurology, Sree Chitra Tirunal Institute for Medical Sciences and Technology, Thiruvananthapuram, India; b Department of Neurology, Sree Chitra Tirunal Institute for Medical Sciences and Technology, Thiruvananthapuram, India; c Department of Imaging Science and Interventional Radiology, Sree Chitra Tirunal Institute for Medical Sciences and Technology, Thiruvananthapuram, India

**Keywords:** Nonstenotic carotid plaques, Embolic stroke of undetermined source, Carotid stenosis

## Abstract

**Introduction:**

Nonstenotic carotid plaques, despite being considered low risk for stroke, are increasingly recognized to contribute to embolic strokes of undetermined source (ESUS). Emerging evidence indicates that rather than the degree of stenosis, high-risk plaque characteristics like plaque irregularity and intraplaque hemorrhage may increase the embolic risk. We analyzed the prevalence and characteristics of nonstenotic carotid plaques in ESUS and their implications for stroke recurrence risk.

**Methods:**

We retrospectively identified 274 patients with ESUS between 2012 and 2022. The computed tomography angiograms were analyzed for nonstenotic carotid plaques (<50% stenosis, as per NASCET criteria) by blinded neuroradiologists. The infarct patterns and plaque characteristics were evaluated. The stroke recurrences at 3 months and 1 year were noted.

**Results:**

The mean age of the study population was 57.99 ± 12.86 years, and 65.3% were males. The nonstenotic plaques (30–50% stenosis) were more common on the symptomatic side of stroke (13.3% vs. 7.3%, *p* = 0.026). Plaques with irregularity were also more frequent on the symptomatic side (16.4% vs. 9.7%, *p* = 0.023). Patients with symptomatic nonstenotic plaques had a higher incidence of embolic (25% vs. 5%, *p* = 0.000) and watershed infarcts (16.7% vs. 4.6%, *p* = 0.005). The stroke recurrence risk and mortality at 3 months and 1 year did not differ between the 2 groups.

**Conclusion:**

In this single-center retrospective study, nonstenotic carotid plaques with 30–50% stenosis were more prevalent on the symptomatic side, suggesting a potential role in stroke mechanism. The higher rates of embolic and watershed infarcts and the presence of plaque irregularity on the symptomatic side support this association.

## Introduction

Embolic strokes of undetermined source (ESUS) constitute a therapeutically relevant clinical entity which is defined as non-lacunar brain infarcts in the absence of proximal arterial stenosis or cardioembolic sources. The causes of ESUS include minor-risk potential cardioembolic sources, covert paroxysmal atrial fibrillation, malignancy, paradoxical embolism, and arteriogenic emboli [1].

Currently only atherosclerotic plaques causing more than or equal to 50% intra- or extracranial stenosis are considered to be causative of stroke [2]. Emerging evidence suggests that a subset of ESUS, is attributable to thromboembolic events originating from undiagnosed plaque disruption in nonstenotic (<50% stenosis) carotid arteries [3]. Several observations support the association between nonstenotic carotid plaques and ESUS. First, nonstenotic plaques are more frequently observed on the carotid ipsilateral to the side of stroke, with larger plaques also more prevalent on that side, despite the weak correlation between plaque size and stenosis [4, 5]. Second, ESUS patients with ipsilateral nonstenotic plaques show a lower prevalence of atrial fibrillation, implying that these plaques contribute to stroke risk [6]. Lastly, high-risk plaque characteristics, particularly intraplaque hemorrhage, are more commonly found on the ipsilateral side of stroke [7, 8]. These findings underscore the critical role of nonstenotic carotid plaques in ESUS. Recent data suggest reclassifying this patient subgroup as having symptomatic nonstenotic carotid disease if the ipsilateral carotid plaque exhibits high-risk features such as intraplaque hemorrhage, a lipid-rich necrotic core, fibrous cap rupture, or ulceration [3, 9].

Evaluating high-risk plaque features in nonstenotic carotid atherosclerosis may prevent unnecessary investigations for ESUS and ensure optimized secondary prevention. These patients may also benefit from a targeted therapeutic approach [10, 11]. We analyzed the prevalence and characteristics of nonstenotic carotid plaques in ESUS and their implications for risk of stroke recurrence.

## Methods

This single-center, retrospective cohort study included patients aged 18 years or older who were admitted with a first-ever acute ischemic stroke meeting the criteria for ESUS between 2012 and 2022 at a comprehensive stroke care unit with available computed tomography (CT) angiography. Patients were diagnosed to have ESUS if the following criteria were satisfied: (1) the stroke is non-lacunar, as confirmed by CT or MRI; (2) the transthoracic echocardiogram is negative; (3) cardiac monitoring is negative; (4) there is no evidence of ≥50% luminal stenosis due to extracranial or intracranial atherosclerosis in the relevant arteries; and (5) no other specific etiology of stroke is identified (e.g., arteritis, vasculitis, dissections, active drug abuse). Patients with strokes in multiple vascular territories, known stroke etiology, or pre-stroke modified Rankin Scale (mRS) >2 were excluded. Evaluation for other potential causes of ESUS included CT angiography to exclude significant extracranial and intracranial atherosclerosis, transthoracic echocardiography for assessment of cardiac sources including PFO, and standardized 7-day cardiac rhythm monitoring for detection of atrial fibrillation. The clinical and demographic details, including risk factors and stroke severity, were collected. Stroke severity was assessed using the National Institutes of Health Stroke Scale (NIHSS). A neuroradiologist blinded to the clinical data reviewed CT angiograms to classify carotid plaques based on high-risk morphological features like plaque length, thickness, irregular surface, calcification, and ulceration.

Calcified plaque was defined as a plaque with more than 50% of its composition consisting of calcified components. On MDCTA, plaque ulceration was defined as the extension of contrast material beyond the vascular lumen into the plaque by at least 1 mm, identified in at least two imaging planes. Plaque surface irregularity was defined as a luminal surface fluctuation measuring 0.3–0.9 mm.

The NASCET criteria were used to calculate the percentage diameter of carotid stenosis. Substenotic carotid disease was defined as carotid stenosis of less than 50%. The pattern of infarcts and the number of acute and chronic infarcts were documented with imaging patterns categorized into territorial, branch occlusion, small superficial embolic, watershed, and perforator patterns to understand the underlying mechanisms of stroke. A representative image has been shown in Figure 1. The primary outcome measure was the relationship of high-risk plaque features to the first-ever AIS. Patients received standard secondary stroke prevention therapy as per institutional stroke management protocols, including antiplatelet therapy and high-intensity statins. In ESUS patients, all received dual antiplatelet therapy for 3 weeks and in patients with substenotic carotid stenosis with high-risk plaque features, 3 months of dual antiplatelet therapy was given. All patients received high-dose statins. Patients were followed up at intervals of 3 months and 1 year to document the occurrence of strokes, transient ischemic attacks, and other vascular events.

**Fig. 1.**
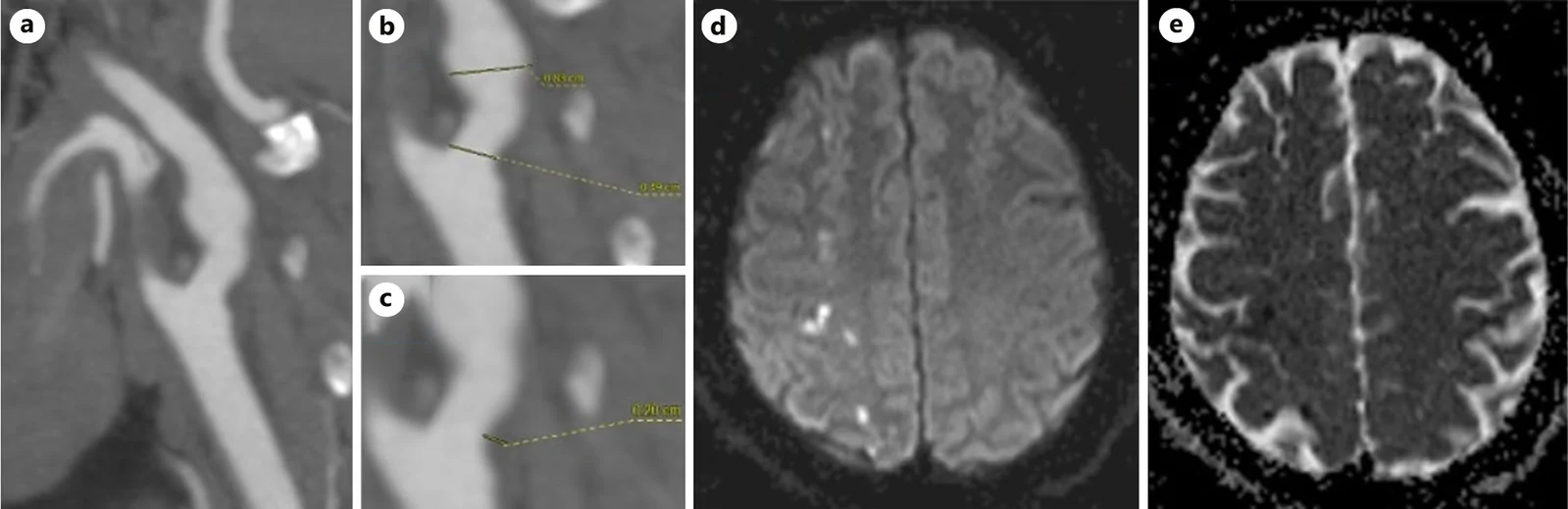
Sagittal reformat of CT angiogram. **a** A hypodense plaque involving the right carotid bulb and proximal cervical ICA, causing ∼40% stenosis with evidence of surface irregularity. **b**, **c** The stenosis percentage and plaque thickness measurements, respectively. Axial DWI (**d**) and corresponding ADC maps (**e**) demonstrate watershed infarcts along the right ACA-MCA and internal watershed territory. CT, computed tomography; ICA, intracranial artery; DWI, diffusion-weighted imaging; ACA-MCA, anterior cerebral artery-middle cerebral artery.

### CT Acquisition and Processing

All CT angiograms were done using the institutional protocol. Brilliance iCT a 256-slice CT scanner (Philips Healthcare, Cleveland, USA) was used for imaging. An infusion of 60 mL of iodinated contrast media (Isovue 370, Bracco Imaging) was given, and image acquisition was done utilizing a bolus tracking method. The source data were then reconstructed into 1 mm axial, 7 mm axial, sagittal, and coronal maximum intensity projection pictures, which were then transferred to a picture archiving and communication system for detailed analysis.

### Statistical Analysis

The statistical analysis involved summarizing categorical variables using frequency analysis, while continuous variables were presented as mean and standard deviation. Comparisons between patients with and without ipsilateral nonstenotic carotid plaques were performed using chi-square tests for categorical variables (such as risk factors, infarct pattern, stenosis status, plaque characteristics, and outcome measures) and independent sample *t* tests for continuous variables (including age, NIHSS score, and baseline investigations). Similarly, comparisons between symptomatic and asymptomatic carotid plaques, based on stenosis status and plaque characteristics, were conducted using chi-square tests and independent sample *t* test.

## Results

Of 560 ESUS patients, 286 were excluded based on criteria and 274 were included in the analysis (Fig. 2). The mean age was 57.99 ± 12.86 years, with 179 (65.3%) males. Diabetes mellitus (51.1%) and hypertension (62%) were the most common vascular risk factors. The mean NIHSS score at admission was 10.55 ± 6.79. Table 1 details the demographic and clinical characteristics.

**Fig. 2.**
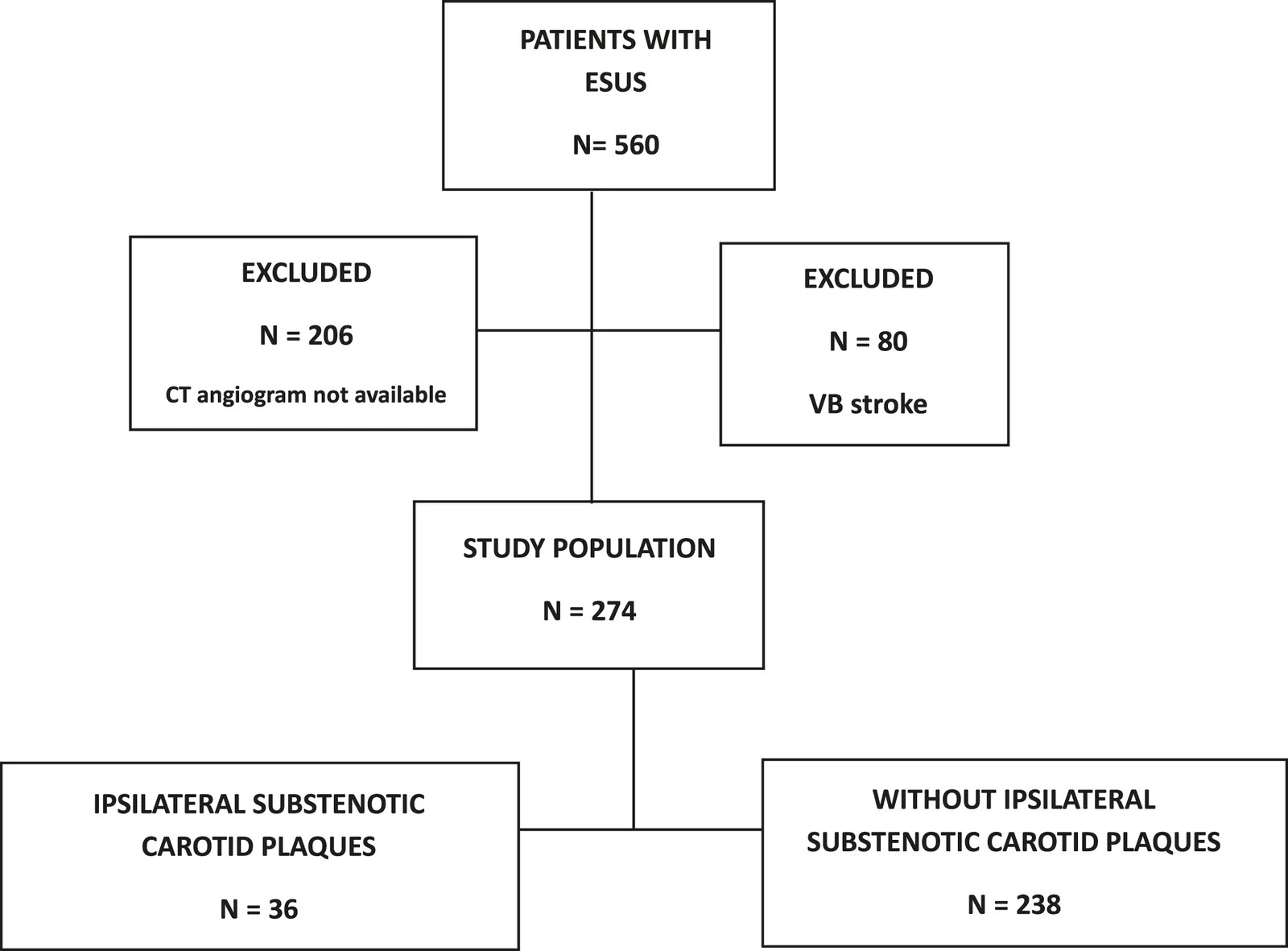
Flowchart showing patient selection and classification based on the presence or absence of ipsilateral substenotic carotid plaques in patients with embolic stroke of undetermined source. Substenotic carotid plaques refers to plaques causing <50% luminal narrowing. ESUS, embolic stroke of undetermined source; CT, computed tomography; VB stroke, vertebrobasilar stroke.

**Table 1.** Clinical and demographic characteristics of study population

Baseline variables	All patients (*n* = 274)
Age, SD, years	57.99±12.86
Male sex, *n* (%)	179 (65.3)
Admission NIHSS (mean±SD)	10.55±6.79
Risk factors, *n* (%)	
Diabetes mellitus	140 (51.1)
Hypertension	170 (62)
Dyslipidemia	123 (44.9)
Smoking	50 (18.2)
Alcohol	53 (19.3)
CAD	48 (17.5)
POVD	2 (0.7)
Investigations	
Admission blood sugar, mg/dL	168.15±79.7464
HbA1C, %	7.12±2.06
Admission systolic BP, mm Hg	152.28±27.99
Diastolic BP, mm Hg	87.28±17.68
Fasting cholesterol, mg/dL	194.96±58.98
HDL, mg/dL	45.52±16.67
LDL, mg/dL	128.40±89.06
TGL, mg/dL	100.50±49.76
ASPECT score (mean±SD)	6.86±2.7
Management, *n* (%)	
IV thrombolysis	63 (29)
Mechanical thrombectomy	29 (10.6)
mRS at 3 months, *n* (%)	
Good outcome (0–2)	137 (50)

Values are expressed as mean ± standard deviation (SD) or number (percentage) as appropriate. ASPECTS, Alberta Stroke Program Early CT Score; BP, blood pressure; CAD, coronary artery disease; HbA1C, glycated hemoglobin; HDL, high-density lipoprotein; IV, intravenous; LDL, low-density lipoprotein; mRS, modified Rankin Scale; NIHSS, National Institutes of Health Stroke Scale; POVD, peripheral occlusive vascular disease; TGL, triglycerides.

Patients with ipsilateral nonstenotic carotid plaques were older (62.8 ± 11.03 vs. 57.26 ± 12.98 years, *p* = 0.016) and had a higher prevalence of hypertension (77.8% vs. 59.7%, *p* = 0.037) compared to those without nonstenotic carotid plaques (Table 2). High-density lipoprotein (HDL) levels were also higher (52 ± 27.74 mg/dL vs. 44.56 ± 14.12 mg/dL, *p* = 0.013) in them. The remaining risk factors, including diabetes, dyslipidemia, coronary artery disease, and biochemical parameters (total cholesterol, low-density lipoprotein, triglycerides), were comparable between the 2 groups.

**Table 2.** Comparison of characteristics between patients with and without ipsilateral nonstenotic carotid plaques

Variable	All patients (*n* = 274)	With ipsilateral nonstenotic carotid plaques (36)	Without ipsilateral nonstenotic carotid plaques (238)	*p* value
Age, (mean±SD), years	57.99±12.86	62.80±11.03	57.26±12.98	**0.016**
Male sex, *n* (%)	179 (65.3)	28 (77.8)	151 (63.4%)	0.135
Admission NIHSS (mean±SD)	10.55±6.79	9.31±5.97	10.74±6.91	0.239
Medical history				
Diabetes mellitus (mean±SD), *n* (%)	140 (51.1)	22 (61.1)	118 (49.6)	0.197
Hypertension, *n* (%)	170 (62)	28 (77.8)	142 (59.7)	**0.037**
Dyslipidaemia, *n* (%)	123 (44.9)	18 (50)	105 (44.1)	0.508
CAD, *n* (%)	48 (17.5)	9 (25)	39 (16.4)	0.205
HBA1C (mean±SD), %	7.12±2.06	7.62±1.74	7.05±2.1	0.123
Admission blood sugar (mean±SD), mg/dL	168.15±79.7464	183±80.24	165.91±79.66	0.232
Admission systolic BP (mean±SD), mmHg	152.28±27.99	157.639±28.98	151.57±27.82	0.219
Admission diastolic BP (mean±SD), mmHg	87.28±17.68	86.17±20.49	87.45±17.27	0.686
Fasting cholesterol (mean±SD), mg/dL	194.96±58.98	206.91±58.23	193.18±59	0.199
HDL (mean±SD), mg/dL	45.52±16.67	52±27.74	44.56±14.12	**0.013**
LDL (mean±SD), mg/dL	128.4±89.06	127.23±53.46	128.58±93.32	0.933
TGL (mean±SD), mg/dL	100.50±49.76	106.77±37.82	99.57±51.315	0.426

Values are presented as mean ± standard deviation (SD) for continuous variables and, number (percentage) for categorical variables. *p* values indicate statistical significance. BP, blood pressure; CAD, coronary artery disease; HbA1C, glycated hemoglobin A1C; HDL, high-density lipoprotein; LDL, low-density lipoprotein; NIHSS, National Institutes of Health Stroke Scale; TGL, triglycerides.

Nonstenotic carotid plaques were more common on the symptomatic side of stroke (13.3% vs. 7.3%, *p* = 0.026) with more irregularity of plaques (16.4% vs. 9.7%, *p* = 0.023) on the symptomatic side (Table 3). The other plaque characteristics, including ulceration, calcification, plaque length, and thickness, showed no significant difference between ipsilateral and contralateral nonstenotic carotid plaques. We did not observe any statistically significant sex-based differences in the prevalence of nonstenotic carotid plaques or high-risk plaque features in our cohort.

**Table 3.** Comparison of plaque characteristics and infarct pattern between symptomatic and asymptomatic carotid plaques

Variable	Symptomatic side	Asymptomatic side	*p* value
Prevalance, *n* (%) [30–50% stenosis]	36 (13.3)	20 (7.3)	**0.026**
<50% stenosis	96 (35%)	76 (27.74)	0.074
Plaque characteristics			
Irregularity, *n* (%)	45 (16.4)	27 (9.7)	**0.023**
Ulceration, *n* (%)	13 (4.7)	12 (4.4)	0.838
Calcification, *n* (%)	42 (15.3)	33 (12)	0.263
Thickness >3 mm, *n* (%)	21 (58.33)	16 (80)	0.799
Thickness >5 mm, *n* (%)	5 (13.89)	4 (20)	0.481
Mean maximum thickness (mm±SD)	3.28±3.35	2.94±1.46	0.458
Pattern of infarct			
Mean maximum length (mm±SD)	12.17±5.79	10.37±5.90	0.987
Territorial, *n* (%)	8 (22.22)	100 (42)	**0.024**
Branch occlusion, *n* (%)	5 (13.9)	44 (18.5)	0.502
Watershed, *n* (%)	6 (16.7)	11 (4.6)	**0.005**
Perforator, *n* (%)	8 (22.22)	56 (23.5)	0.863
Embolic, *n* (%)	9 (25)	12 (5)	**0.000**

Values are presented as mean ± standard deviation (SD) for continuous variables and, number (percentage) for categorical variables.

On analyzing the pattern of the infarct, embolic (25% vs. 5%, *p* = 0.0001) and watershed (16.7% vs. 4.6%, *p* = 0.005) infarcts were more frequent in patients with ipsilateral nonstenotic carotid plaques, while territorial infarcts were more common in those without ipsilateral nonstenotic carotid plaques (22.2% vs. 42%, *p* = 0.024) (Table 3). Large vessel occlusion was less frequent in patients with ipsilateral nonstenotic plaques (72.2% vs. 52.1%, *p* = 0.017), and M1 MCA occlusion was significantly lower in this group (13.9% vs. 29.8%, *p* = 0.032).

At 3 months, good functional outcomes (mRS 0–2) were similar in both patients with and without ipsilateral nonstenotic carotid plaques (55.6% vs. 50.4%, *p* = 0.567) (Table 4). The stroke recurrence remained low and comparable at 3 months (2.8% vs. 0.8%, *p* = 0.319) and 1 year (2.8% vs. 2.5%, *p* = 0.915) in both the groups. Among 247 patients followed for 1 year, good outcomes (mRS 0–2) were similar in patients with and without ipsilateral nonstenotic carotid plaques (62.5% vs. 67%).

**Table 4.** Comparison of outcome between patients with and without ipsilateral nonstenotic carotid plaques

Outcome	With ipsilateral nonstenotic carotid plaques (36)	Without ipsilateral nonstenotic carotid plaques (238)	*p* value
mRS at 3 months, *n* (%)			
Good outcome (0–2)	20 (55.6)	117 (50.4)	0.595
Bad outcome (3–6)	16 (44.4)	115 (49.6)	​
mRS at 1 year, *n* (%)			
Good outcome (0–2)	20 (62.5)	144 (67)	0.377
Bad outcome (3–6)	12 (37.5)	71 (33)	​
Stroke recurrence at 3 months	1	2	0.319
Stroke recurrence at 1 year	1	6	0.915
Death at 3 months	0	7	0.291
Death at 1 year	1	8	0.867

mRS, modified Rankin Scale. A good outcome is defined as mRS score 0–2; a bad outcome is defined as mRS score 3–6.

## Discussion

This study highlights the potential role of nonstenotic carotid plaques in ESUS. We found that nonstenotic carotid plaques with high-risk features like plaque irregularity were more prevalent on the symptomatic side and were associated with embolic and watershed infarcts.

The mean age of the study population was 57 years, which is similar to other stroke registries from India and 10 years younger than the mean age of the population from the developed countries [12]. Hypertension (62.0%) and diabetes mellitus (51.1%) were the most common risk factors, mirroring regional prevalence [13]. Age showed a significant independent association with the presence of nonstenotic carotid plaques, indicating its role in atherosclerosis and increased stroke risk with advancing age.

Hypertension was significantly more prevalent among individuals with nonstenotic carotid plaques, emphasizing its association with carotid atherosclerosis. Hypertension contributes to atherosclerosis through endothelial dysfunction, hemodynamic stress, and metabolic changes, promoting plaque formation and thickening of arterial walls. Interestingly, the study revealed higher HDL levels in patients with these nonstenotic plaques, defying the conventional expectation of lower HDL in the face of atherosclerosis risk [14]. This suggests that it may be a combination of risk factors which may be responsible for atherosclerosis in an individual patient.

The study highlighted important insights into carotid plaque characteristics and their implications for stroke risk assessment. Notably, nonstenotic plaques with 30–50% stenosis were more common on the ipsilateral side of stroke, suggesting a potential role in embolic stroke mechanisms, even without severe stenosis. This aligns with previous studies highlighting the role of emboli originating from nonstenotic plaques in causing ischemic strokes, underscoring the importance of thorough evaluation and management of these lesions in clinical practice [5]. Though 13% percent patients exhibited nonstenotic carotid plaques on the symptomatic side compared to seven percent on the asymptomatic side, this was less than the prevalence of the nonstenotic carotid plaques in the meta-analysis by Ospel et al. [15] (overall 51%, 48% ipsilateral, 55% in ESUS). Another study by Ntaios et al. [16] reported a 38.6% prevalence and found that atrial fibrillation is detected less frequently in ESUS patients with nonstenotic carotid plaques than in those without them.

A CT angiogram was available in all our patients as non-contrast head CT and head-and-neck CT angiograms are routinely performed in all patients with AIS. The plaque irregularity was significantly higher on the symptomatic side of nonstenotic carotid stenosis in our study, and this was statistically significant even after adjusting for the degree of stenosis and plaque thickness, indicating vulnerability to plaque rupture and inflammatory processes. This is similar to the study by Singh et al. and De Weert et al. [17, 18]. This reiterates the fact that in patients with ischemic stroke irrespective of the percentage of the carotid stenosis, the plaque characteristics are important in assessing its role in the etiology of the stroke.

In our study, no statistically significant difference was found in the prevalence of plaque ulceration or calcification between symptomatic and asymptomatic carotid stenosis. The study by De Weert et al. [18] demonstrated that 24% of patients with at least one atherosclerotic carotid artery had ulcerations. Most of these (69%) were located proximal to the point of maximum stenosis, with ulcer types 1 and 3 being the most frequently observed. The prevalence of ulcerated plaques was less in our study compared to previous studies. However, the link between these features and ipsilateral strokes is weak, with many other studies failing to show a significant association [15]. This could be due to the relatively low tissue contrast of the CT angiogram, which hampers accurate differentiation of various plaque components [19]. Additionally, the nonsignificant findings might reflect the inherent variability in plaque characteristics and the influence of other factors such as the interval between symptom onset and imaging [20].

The maximum carotid plaque length and thickness did not significantly differ between the symptomatic and asymptomatic sides. Similarly, the number of plaques exceeding 3 mm and 5 mm showed no significant differences. In a study by Coutinho et al. [5] involving 85 ESUS patients, ipsilateral stroke was significantly associated with plaque thicknesses of ≥5 mm. However, our study did not replicate these findings. In another study of 152 ESUS patients by Jumah et al. [21], high-risk plaque features, such as plaque thickness >3 mm and plaque length >1 cm, were more commonly found on the symptomatic side compared to the contralateral side. In addition, previous studies with CT angiogram have observed good interobserver agreement on plaque thickness measurements, demonstrating excellent reproducibility [5, 22]. Though in our study the plaque characteristics were assessed by an experienced neuroradiologist, we did not find any difference in the plaque length and thickness between the 2 groups.

While plaque thickness is a useful marker of stroke risk, plaque volume may provide a more comprehensive assessment of atherosclerotic burden and plaque vulnerability [20]. However, plaque volume measurement is technically challenging and lacks standardized methods. Overall, CT angiography plaque characteristics such as surface irregularity, ulceration, hypodensity, and calcification have shown inconsistent associations with ipsilateral stroke in ESUS [8, 17, 20, 21, 23]. Ultra-sonogram-based studies suggest that plaque thickness and size may be more important markers of culprit lesions in ESUS [24, 25].

Plaque irregularity likely reflects plaque instability and vulnerability, which may predispose to artery-to-artery embolism even in the absence of hemodynamically significant stenosis. Surface irregularity may indicate endothelial disruption, inflammation, plaque remodeling, or superimposed thrombus formation, thereby increasing embolic potential. In contrast, plaque size or thickness may reflect overall atherosclerotic burden rather than plaque vulnerability. Although plaque ulceration is also considered a marker of vulnerable plaque, it was not significantly associated with ESUS in our cohort. This may be related to the relatively low frequency of ulcerated plaques, limited statistical power, or difficulty in detecting small ulcerations on CTA. Plaque irregularity may, therefore, represent a more sensitive imaging marker of plaque instability in nonstenotic carotid disease. In our study, only plaque irregularity was associated with ESUS.

Patients with ipsilateral nonstenotic carotid plaques in our study had significantly higher rates of embolic infarcts compared to those without such plaques. Conversely, territorial infarcts were more common in patients without these plaques. Nonstenotic carotid plaques may elevate stroke risk by harboring embolic materials, such as cholesterol crystals or thrombi, which can embolize to various brain regions. Even without significant luminal narrowing, these plaques can disrupt local hemodynamics or act as sources of microemboli, particularly affecting vulnerable areas like watershed zones. The lower rates of major vessel occlusion in patients with nonstenotic carotid plaques suggest that these plaques may cause strokes by producing smaller thrombi. In contrast, patients with ESUS without nonstenotic carotid plaques may have occult cardiac sources of embolism, resulting in larger emboli that lead to major vessel occlusions and territorial infarcts.

At the 3-month follow-up, the occurrence of recurrent strokes was comparable between the two groups, with 1 patient experiencing recurrence in the nonstenotic plaque group and 2 patients in the group without such plaques. Similarly, at the 1-year follow-up, 1 patient with nonstenotic carotid plaques had a recurrent stroke compared to 6 patients without these plaques. These findings suggest that while ipsilateral nonstenotic carotid plaques did not significantly influence short-term or long-term stroke recurrence rates in this cohort, the observed occurrences underscore the ongoing risk of stroke in both groups. The low risk of recurrence in our cohort may be because these patients were treated at comprehensive stroke centers and received intensive medical management with dual antiplatelets and high-dose statins.

A recent meta-analysis by Singh et al. [17] demonstrated that symptomatic, nonstenotic carotid plaques (less than 50%) with high-risk features significantly increase the risk of recurrent ipsilateral stroke or transient ischemic attacks. This risk is comparable to that observed in stenotic carotid plaques (greater than 50%) as reported by NASCET [26] and ECST [27], which showed rates of approximately 9% and 4% per year, respectively. Another retrospective observational study showed that at 3-year follow-up, the cumulative incidence of recurrent ipsilateral stroke in patients with symptomatic nonstenotic carotid plaque was 7.4% [28]. This reinforces the importance of identifying the nonstenotic plaques so that strict risk factor control and intensive medical management can reduce the long-term risk too. In patients with recurrent ipsilateral ischemic events despite optimal medical therapy, carotid endarterectomy or carotid artery stenting may be considered on an individual basis.

The strength of the study is that the patient cohort strictly fulfills the criteria for ESUS. A neuroradiologist and a neurologist independently reviewed all study images in a blinded fashion. To enhance study validity, we defined nonstenotic carotid plaques as those with 30–50% stenosis, excluding potentially insignificant, very small plaques. However, the study has limitations. The sample size was relatively small, and numerous patients with ESUS who underwent MR angiography alone for stroke etiology evaluation were excluded from the analysis.

While the vascular imaging features are important in ESUS evaluation, they should not exclude investigating other embolic stroke causes. Grosse et al. found nonstenosing carotid plaques associated with left atrial disease and atrial fibrillation, highlighting the need for thorough cardiac evaluation [29]. Additionally, evidence suggests many ESUS patients may have multiple embolic sources, underscoring the importance of comprehensive assessment beyond plaque features [30].

There is a role for carotid plaque imaging in patients with nonstenotic carotid plaques to identify the high-risk plaque features like lipid-rich necrotic core and intraplaque hemorrhage which will further reinforce the role of these plaques in the etiology of stroke.

## Conclusion

Nonstenotic carotid plaques with 30–50% stenosis were more prevalent on the symptomatic side, suggesting a potential stroke etiology in ESUS patients. The higher rates of embolic and watershed infarcts and plaque irregularity in this subgroup reinforce this to be a possible stroke etiology. With the best medical management, the risk of stroke recurrence was low in patients with nonstenotic carotid plaques. Careful evaluation for the presence of high-risk plaque features in the ipsilateral nonstenotic carotid plaque using advanced imaging techniques may be needed in patients with ESUS.

## Statement of Ethics

The study involved human participants and was conducted in accordance with the Declaration of Helsinki. The study protocol was reviewed and approved by the Institutional Ethics Committee, Sree Chitra Tirunal Institute for Medical Sciences and Technology, Trivandrum, with Approval No. SCT/IEC/1969/November/2022, dated February 27, 2023. Written informed consent was waived by the Institutional Ethics Committee as the data were collected retrospectively.

## Conflict of Interest Statement

P.N. Sylaja was a member of the journal’s Editorial Board at the time of submission. The authors declare no conflict of interest.

## Funding Sources

The authors did not receive any funding for the conduct of this study.

## Author Contributions

Conceptualization, data curation, and project administration were carried out by M.N. and S.P.N. Supervision of the study was carried out by M.N. The data were collected by M.N. and A.A. The formal analysis of the data was done by A.D. The original draft was written by M.N., A.V., and S.P.N. Reviewing and editing of the manuscript was done by M.N., A.V., S.E.S., A.A., and S.K.

## Data Availability

All data generated or analyzed during this study are included in this article. Further inquiries can be directed to the corresponding author.
